# Association between physical activity and menopausal symptoms in perimenopausal women

**DOI:** 10.1186/1472-6874-14-122

**Published:** 2014-10-03

**Authors:** Min-Ju Kim, Juhee Cho, Younjhin Ahn, Gyeyoon Yim, Hyun-Young Park

**Affiliations:** Division of Cardiovascular and Rare Diseases, Center for Biomedical Science, Korea National Institute of Health, 187 Osongsaengmyeng 2-ro, Osong-eup, Heungdeok-gu, Cheongju-si, Chungbuk, 361-951 Korea; Department of Epidemiology, Johns Hopkins Bloomberg School of Public Health, Baltimore, MD USA; Department of Health Sciences and Technology, Samsung Advanced Institute for Health Sciences and Technology, Sungkyunkwan University, Seoul, Korea

**Keywords:** Menopause-specific Quality of Life, Physical activity, Perimenopause

## Abstract

**Background:**

Physical activity may be an effective way of preventing or attenuating menopause-related symptoms, and it has been shown to improve quality of life in menopausal women. However, there have been some inconsistencies regarding between exercise and menopausal symptoms, and study investigating this association has been scarce in Korea. In this study, the association between physical activity and menopausal symptoms in perimenopausal women in Korea was assessed.

**Methods:**

This cross-sectional observational study was conducted between November 2012 and March 2013. In total, 2,204 healthy women aged 44–56 years were recruited from a healthcare center at the Kangbuk Samsung hospitals for investigating women’s attitudes towards menopause. To investigate the influence of physical activity on perimenopause-associated symptoms, 631 perimenopausal women were selected for this study. Their physical activity levels were assessed using the International Physical Activity Questionnaire (IPAQ) short form. The Menopause-specific Quality of Life (MENQOL) questionnaire was used to assess menopause-related symptoms.

**Results:**

The study participants were, on average, 48.5 ± 2.7 years old and had a mean body mass index of 22.8 ± 3.1 kg/m^2^. The total MENQOL score and the psychosocial and physical subscores exhibited U-shaped trends in relation to the level of physical activity. Multiple linear regression analysis adjusted for confounding variables showed that perimenopausal women who performed moderate physical activity reported significantly lower psychosocial (β = -0.413, *P* = 0.012) and physical symptoms (β = -0.445, *P* = 0.002) than women who performed low physical activity. By contrast, a high level of physical activity did not influence the MENQOL total score and subscores relative to the low activity group. In addition, no associations were observed between physical activity and the vasomotor and sexual symptoms in any group.

**Conclusions:**

Moderate level of physical activity was associated with reduced psychosocial and physical menopause symptoms in perimenopausal Korean women. Although these findings must be confirmed by prospective longitudinal studies, they suggest that physical activity may improve the symptoms of menopause, thereby increasing quality of life.

**Electronic supplementary material:**

The online version of this article (doi:10.1186/1472-6874-14-122) contains supplementary material, which is available to authorized users.

## Background

Menopause is a physiological phase that is characterized by the permanent cessation of menstrual periods in women due to loss of ovarian follicular function [1]. During the menopausal transition, women experience various physical, psychological, and social changes that may affect their quality of life [2]. Several symptoms, including hot flushes, night sweats, vaginal dryness, depression, irritability, headache, and sleep disturbance, can occur more frequently in this period [3]. In the Menopause-specific Quality of Life (MENQOL) questionnaire, these symptoms are divided into four domains, namely, the physical, vasomotor, psychosocial, and sexual symptom domains [4].

Hormone replacement therapy can be used to alleviate menopausal symptoms [5], but given the possible serious adverse effects of hormone therapy, many women are searching for alternative therapies to reduce their menopausal symptoms [5, 6]. One such therapy is exercise, which is one of the most commonly used alternatives for menopausal symptoms [5]. Physical activity is also associated with many health benefits, including a decreased risk of cardiovascular disease, metabolic syndrome, obesity, cancer, osteoporosis, and depression [7]. There is evidence that regular physical activity may be an effective way of preventing or attenuating menopause-related symptoms [3]. Several previous studies showed that physical activity significantly reduces menopausal symptoms [8, 9], but other studies have found that physical activity improves general symptoms such as physical and psychosocial symptoms, although it does not influence specific symptoms such as vasomotor and sexual symptoms [10–13]. A meta-analysis has reported inconsistent results regarding the effect of physical activity on menopausal symptoms, with mixed results being observed for different types of symptoms [14]. In addition, engaging in habitual physical activity at least 60 minutes/day showed favorable effects on the prevention of menopausal symptoms, and a high total physical activity level was also associated with less climacteric symptoms [15, 16]. Previous study in multiethnic groups of midlife women showed that the specific types of women’s physical activity influenced the prevalence and severity of menopausal symptoms, which was differed by ethnicity [17].

Although evidence suggests the benefits of physical activity on menopausal symptoms, there have been some inconsistencies regarding between exercise and menopausal symptoms. In addition, study investigating this association has been scarce in Korea. In the present study, the association between physical activity and the menopausal symptoms of perimenopausal women in Korea was investigated. The amount of activities performed was used rather than specific type of activities.

## Methods

### Ethics statement

The study was approved by the Institutional Review Board of the Kangbuk Samsung hospital (IRB No. KBC12156). Written informed consent was obtained from all subjects before they participated in the study.

### Study participants

The Kangbuk Samsung Health Study is a cohort study of South Korean men and women aged 18 years or older who underwent a comprehensive annual or biennial health examination at the clinics of the Kangbuk Samsung Hospital Total Healthcare Center in Seoul and Suwon, South Korea. This ancillary study was a cross-sectional observational study and its purpose was to investigate the attitudes of Korean women towards menopause. Among the middle-aged women who visited a healthcare center between November 2012 and March 2013, the subjects who agree to participate in this study were selected. The participation rate was about 71%. Inclusion criteria were age of 44-56 years, no serious illness, and the ability to understand a questionnaire. Subjects who were diagnosed with cancer and were being treated were excluded in the screening stage. In total, 2,204 healthy women were invited to participate in the study. The participants were divided into three groups according to menopausal status on the basis of Stages of Reproductive Aging Workshop (STRAW) criteria, as follows: premenopause is defined as having regular menstrual periods; perimenopause is characterized by persistent ≥ 7 days difference in length of consecutive cycles or interval of amenorrhea of ≥ 60 days; and postmenopause is the period after 12 consecutive months of amenorrhoea [18]. To investigate the influence of physical activity on perimenopause-associated symptoms, the 732 perimenopausal women were selected. Fifty subjects who had a history of hormone replacement therapy for the management of menopausal symptoms, 38 with missing data on their MENQOL questionnaire, 12 who responded ‘no symptoms’ for all items of the MENQOL, and one with missing information on physical activity were excluded from the analysis. Therefore, 631 women were eligible for this study.

### Measurements

Body weight and height were measured to the nearest 0.1 kg or 0.1 cm and body mass index (BMI) was calculated as body weight (kg) divided by height (meters) squared. Waist circumference (WC) was measured at the midpoint between the lower ribs and the top of the iliac crest in the standing position. Blood pressure (BP) was measured three times with a Welch Allyn sphygmomanometer after a 5 min rest period and the average systolic and diastolic BP values of two measurements were calculated.

Blood samples were collected after at least a 10 h fast. Fasting plasma glucose (FPG), total cholesterol (TC), triglyceride (TG), and high-density lipoprotein cholesterol (HDL-C) levels were measured enzymatically (Module Extention D2400, Roche, Japan). Hemoglobin A_1C_ was evaluated using an immunoturbidimeter (Integra 800, Roche, Switzerland). The homeostatic model for insulin resistance (HOMA-IR) was calculated using the following formula: fasting insulin (μIU/mL) × fasting glucose (mg/dL)/(22.5 × 18).

Current smoking status was categorized as yes or no. Respondents who reported being married or cohabiting were categorized as living with a partner. Those reporting that they had never married or were separated, divorced or widowed were categorized as living without a partner. Family income was classified as < 4 million won and > 4 million won per month. Education level was categorized as high school or lower, or college/university. Parity was characterized as 1–2 children and 3 or more children.

### Menopausal symptoms

The MENQOL questionnaire was used to assess menopause-related symptoms [19]. This questionnaire consists of 29 items in four domains, namely, vasomotor (three items), psychosocial (seven items), physical (16 items) and sexual (three items), and the subjects were asked to indicate whether they had experienced each symptom and, if they had, to rate the symptom according to its severity from 0 (not at all bothered) to 6 (extremely bothered) [16]. For data analysis, this seven-point Likert scale was converted to a score ranging from 1 (not experiencing a symptom) to 8 (extremely bothered) [16]. The mean score of each domain was calculated separately. In order to d emonstrate the internal consistency of the four domains in the MENQOL, Cronbach’s alpha coefficients were calculated. The coefficients were 0.81 for the vasomotor domain, 0.87 for the psychosocial domain, 0.88 for the physical domain, and 0.84 for the sexual symptoms domain, indicating that this scale had an acceptable reliability.

### Physical activity assessment

The physical activity of the women was assessed using the International Physical Activity Questionnaire (IPAQ) short form [20]. This questionnaire asks about three specific types of activity, namely, walking, moderate-intensity activities, and vigorous-intensity activities [20]. The minutes spent every week on each type of activity are computed separately by multiplying the duration and frequency of activity [20]. A continuous activity score is calculated by multiplying the selected metabolic equivalent (MET) value and weekly minutes of activity, therefore expressing physical activity as MET-min per week [20]. The subjects were divided into low, moderate and high levels of physical activity on the basis of their total physical activity (MET-min/week) and the frequency of the activities [20]. The MET values and the level of physical activity were calculated according to the guidelines for data processing and analysis of the IPAQ [20].

### Statistical analysis

Distribution testing for normality was performed using the Shapiro-Wilk test and the data were log-transformed to obtain normalized distributions. The geometric means of log-transformed variables were back-transformed for ease of interpretation and were expressed with 95% confidence intervals (CIs). The baseline characteristics of the study participants were expressed as mean ± standard deviation (SD), geometric mean (95% CI), or number (%). The low, moderate, and high physical activity groups were compared using one-way analysis of variance (ANOVA) with Dunnett’s multiple comparison test for continuous variables and chi-square tests for categorical variables. The relationships between baseline characteristics and physical activity level were assessed using a linear-by-linear association test. The relationships between MENQOL total score/subscores and physical activity level were assessed using ANOVA with Dunnett’s multiple comparison test. Because of missing data on marital status, family income and education, only 476 of 631 participants were included in the main analysis. The associations between physical activity and MENQOL total score/subscores were assessed by multiple linear regression analysis. MENQOL total score/subscore served as dependent variables, while physical activity level served as an independent variable. Adjustments were made for age, BMI, marital status, family income, education, and parity. The strength of the association between the total MENQOL score/subscores and the physical activity level was expressed as the beta coefficient and *P*-values. To assess the clinical significance of the result, the effect size was calculated [21, 22]. *P-*values of <0.05 were considered to indicate statistical significance. All data were analyzed using SPSS Statistics 21 (SPSS Inc., IBM Corp., Chicago, IL, USA).

## Results

### Baseline characteristics

The baseline characteristics of all study participants according to physical activity levels are shown in Table 1. The subjects were, on average, 48.5 ± 2.7 years old. Their mean BMI was 22.8 ± 3.1 kg/m^2^. In total, 57.8%, 28.1%, and 14.1% of the subjects performed low, moderate, and high levels of physical activity, respectively. The women with moderate physical activity were significantly younger than the women in the low physical activity group (*P* = 0.011). Most women were nonsmokers (96.9%), were married or cohabitated (93.6%), had a high family income (84.4%), were highly educated (63.6%), and had 1–2 children (81.3%). The three physical activity groups significantly differed in terms of smoking and marital status (*P* for trend = 0.032 and 0.012, respectively). Compared to the women in the low and high physical activity groups, the women with a moderate level of physical activity were more likely to be living with a partner, to have a high family income, and to be highly educated. The WC, fasting insulin levels, HOMA-IR, and TG levels decreased significantly as the physical activity increased (data not shown) (*P* for trend = 0.040, 0.001, 0.006, and 0.012, respectively). The HDL-C value increased as physical activity increased (data not shown) (*P* for trend = 0.003). The highly active group had significantly lower levels of fasting insulin, HOMA-IR and TG values and higher levels of HDL-C than the low activity group (data not shown) (*P* = 0.002, 0.012, 0.024, and 0.006, respectively).

**Table 1:** **Demographic characteristics of the study population**

Variables	Total (n = 631)	Physical activity level			*P*for trend
		Low (n = 365)	Moderate (n = 177)	High (n = 89)	
Age, y	48.5 ± 2.7	48.7 ± 2.8	48.0 ± 2.6^*^	48.8 ± 2.6	0.656
BMI, kg/m^2^	22.8 ± 3.1	23.0 ± 3.1	22.5 ± 3.0	22.6 ± 2.9	0.257
WC, cm^a^	78.3 (77.5–79.0)	79.0 (78.3–79.8)	77.5 (75.9–78.3)	76.7 (75.2–78.3)	0.040
Smoking, n (%)	14 (3.1)	13 (4.9)	0(0.0)	1 (1.6)	0.032
Marital status, n (%)^b^					0.012
Nevermarried/separated/divorced/widowed	39 (6.4)	31 (8.9)	5 (2.9)	3(3.6)	
Married/cohabitating	568 (93.6)	317 (91.1)	170 (97.1)	81 (96.4)	
Family income, n (%)^c^					0.059
Less than 4 million won	79 (15.6)	58 (19.7)	9 (6.3)	12 (17.1)	
More than 4 million won	429 (84.4)	236 (80.3)	135 (93.8)	58 (82.9)	
Education, n (%)^d^					0.097
High school or lower	215 (36.4)	140 (41.4)	42 (24.9)	33 (39.8)	
College/university	375 (63.6)	198 (58.6)	127(75.1)	50 (60.2)	
Parity, n (%)					0.224
1–2	491 (81.3)	273 (78.7)	147 (87.0)	71 (80.7)	
≥ 3	113 (18.7)	74 (21.3)	22 (13.0)	17 (19.3)	

The data are expressed as mean ± standard deviation, geometric means (95% confidence interval (CI)), or number (%). The physical activity groups were compared using one-way ANOVA for continuous variables and chi-square tests for categorical variables.

*Significantly different from the control group (Low) at p < 0.05 (Dunnett’s test).

^a^Log-transformed values were used for analysis; the geometric means and 95% CIs were back-transformed.

^b^Marital status information was available for 607 participants.

^c^Family income information was available for 508 participants.

^d^Education information was available for 590 participants.

BMI, body mass index; WC, waist circumference.

### Scores for each MENQOL domain

Table 2 shows the mean scores for each MENQOL domain in the three physical activity groups. Physical activity was significantly associated with the total MENQOL score (*P* = 0.027) and specifically the psychosocial and physical subscores (*P* = 0.015 and 0.002, respectively). The moderately active group had the lowest psychosocial and physical subscores, followed by the highly active group and the low activity group; thus the relationship between the psychosocial and physical subscores and physical activity had a U-shaped trend. The relationship between the total MENQOL score and physical activity also exhibited a U-shaped trend (3.1 ± 1.3, 2.8 ± 1.2, and 3.0 ± 1.3 for the low, moderate, and high groups, respectively). However, physical activity level was not significantly associated with the vasomotor or sexual subscores.

**Table 2:** **Relationship between Menopause-specific Quality of Life (MENQOL) total score/subscores and physical activity levels**

MENQOL domains	Total (n = 631)	Physical activity level			*P*-value
		Low (n = 365)	Moderate (n = 177)	High (n = 89)	
Vasomotor	2.3 ± 1.6	2.3 ± 1.6	2.2 ± 1.4	2.2 ± 1.5	0.367
Psychosocial	3.1 ± 1.5	3.3 ± 1.5	2.9 ± 1.4^*^	3.2 ± 1.6	0.015
Physical	3.5 ± 1.3	3.6 ± 1.3	3.2 ± 1.2^*^	3.4 ± 1.4	0.002
Sexual	3.3 ± 1.8	3.4 ± 1.9	3.1 ± 1.8	3.1 ± 1.8	0.249
Total	3.0 ± 1.3	3.1 ± 1.3	2.8 ± 1.2^*^	3.0 ± 1.3	0.027

The data are expressed as mean ± standard deviation and were tested by one-way ANOVA.

*Significantly different from the control group (Low) at p < 0.05 (Dunnett’s test).

Higher MENQOL total scores/subscores indicate worse symptoms (the scores range from 1 to 8).

### Multiple linear regression analysis of MENQOL scores

The results of the multiple linear regression analysis of the MENQOL total score/subscores are presented in Table 3. Among the 631 participants, 476 were included in this main analysis. Compared to the low activity group, women with a moderate level of physical activity had significantly lower MENQOL total scores (β = -0.306, *P* = 0.028). Specifically, these women reported significantly lower psychosocial (β = -0.414, *P* = 0.012) and physical symptoms (β = -0.446, *P* = 0.002) than the low physical activity group after adjustment for age, BMI, marital status, family income, education and parity. By contrast, a high level of physical activity did not influence the MENQOL total score and subscores relative to the low activity group. In addition, no associations were observed between physical activity and the vasomotor and sexual symptoms in any group.

**Table 3:** **Multiple linear regression analysis of the relationship between Menopause-specific Quality of Life (MENQOL) total score/subscores and physical activity levels**

Variables	Total			Vasomotor			Psychosocial			Physical			Sexual		
	β	SE	*P*-value	β	SE	*P*-value	β	SE	*P*-value	β	SE	*P*-value	β	SE	*P*-value
Physical activity															
Low	Reference			Reference			Reference			Reference			Reference		
Moderate	−0.306	0.139	0.028	−0.054	0.173	0.754	−0.414	0.164	0.012	−0.446	0.144	0.002	−0.309	0.196	0.116
High	−0.190	0.173	0.271	−0.154	0.214	0.473	−0.053	0.203	0.795	−0.184	0.179	0.304	−0.369	0.243	0.129

Beta coefficients and P-values are presented.

The regression analysis was adjusted for age, body mass index, marital status, family income, education and parity.

## Discussion

The purpose of this study was to examine the relationship between physical activity and the self-reported vasomotor, psychosocial, physical, and sexual symptoms of perimenopausal women in Korea. The total MENQOL score and the psychosocial and physical subscores exhibited U-shaped trends in relation to the level of physical activity. A moderate level of physical activity was associated with reduced psychosocial and physical symptoms but not with changes in vasomotor and sexual symptoms.

In terms of the severity of menopausal symptoms, as assessed by the Menopause Rating Scale (MRS), Latin Americans reported higher scores in the somatic, psychological and urogenital domains than Europeans and Asians; thus Europeans and Asians appear to report fewer menopausal symptoms [23]. In our sample of 631 perimenopausal women, the highest MENQOL subscore was the physical domain subscore (3.5 ± 1.3), followed by the sexual (3.3 ± 1.8), psychosocial (3.1 ± 1.5), and vasomotor (2.3 ± 1.6) domain subscores. The mean MENQOL total score of our subjects was similar to that of perimenopausal women in Chile [24] and slightly higher than that in perimenopausal women in China and Thailand [25, 26]. Additional file 1 shows the frequency of individual menopausal symptoms in our subjects.

The present study showed that after adjusting for confounding variables, moderate activity was significantly associated with improved psychosocial and physical symptoms but did not associate with changes in vasomotor and sexual symptoms. Although 155 participants with missing data for marital status, family income and education were excluded in the main analysis, Additional file 2 showed that the exclusion of those participants was not problematic. As shown in Additional file 2, the mean scores of each MENQOL domain were similar between 476 participants with complete data and 155 with missing data, indicating no significant difference between each groups of physical activity. Our results are consistent with those of several previous studies, which showed that physical activity correlates inversely with psychological and physical symptoms but not with vasomotor and sexual symptoms [10–13]. In addition, intervention study indicated that the habitual physical activity of at least 60 minutes/day had a favorable influence on the menopausal symptoms, particularly in the psychological and social domains, and other cross-sectional studies have demonstrated that the menopausal women with a low physical activity level reported severe menopausal symptoms [15, 16]. Observational studies consistently show that physical activity associates with mental health problems such as anxiety, stress, and depressive symptoms [12, 27]. A cross-sectional study from Finland found that compared to women with a sedentary lifestyle, physically active women reported significantly fewer somatic symptoms [28]. Furthermore, in the Australian Longitudinal Study on Women’s Health, increased physical activity was associated with decreases in somatic symptoms [29]. This association between physical activity and psychosomatic symptoms may be mediated by several psychological and physiological mechanisms, including diversion from stressful stimuli, improved self-efficacy, enhanced brain aminergic synaptic transmission, and increased levels of endorphins [30].

Recent study in multiethnic groups of midlife women reported that household activity was positively associated with the prevalence and severity scores of psychological menopausal symptoms in both non-Hispanic Asian and non-Hispanic African American women, whereas there was an inverse relationship between increased sports/exercise activity level and the severity scores of the physical symptoms among Hispanics and non-Hispanic Whites [17]. The differences between the ethnic groups with regard to their menopausal symptoms may be explained by sociodemographic, economic and cultural differences as well as different measurement instruments [31]. As our study focused on the amount of activities performed rather than specific type of activities, further examination of the effect of diverse type of physical activities on menopausal symptoms might be needed.

We did not observe an association between physical activity and vasomotor symptoms. Similarly, more than half of the many (>30) studies on this topic have failed to detect a significant association between these variables [14]. In addition, the largest study found that moderate-intensity aerobic exercise did not significantly alleviate vasomotor symptoms [32]. We also did not observe an association between physical activity and sexual symptoms. Although one study has reported that physical activity improves sexual symptoms during the menopausal transition, the relationship between physical activity and sexual symptoms has not been well studied [3]. To our knowledge, most of the studies on this topic focus on menopausal women [10–13, 27–29]: only two studies show limited data on the impact of physical activity on the menopausal symptoms of perimenopausal women [3, 14].

Interestingly, our study indicated that the relationship between physical activity and menopausal symptoms had a U-shaped trend, and moderate level of physical activity was significantly associated with reduced psychosocial and physical symptoms. The effect of physical activity was not dose dependent since the highly active group experienced more symptoms than the moderately active groups. In addition, there was 0.4 point difference in scores for the psychosocial and physical subscores between low and moderate group (3.3 vs. 2.9, *P* = 0.009; 3.6 vs. 3.2, *P* = 0.001), and 0.3 point difference in score for the total MENQOL score (3.1 vs. 2.8, *P* = 0.015). The effect sizes of physical activity on psychosocial, physical and total menopausal symptoms were 0.27, 0.31, and 0.24, respectively. This is certainly nontrivial because effect size of 0.2 generally regarded the threshold for even a small effect [22]. Although whether the relationship between physical activity and menopausal symptoms has clinical significance is questionable, it is estimated that the increasing low to moderate level of physical activity would result in 0.3 or 0.4 point decrease in total MENQOL, psychosocial, and physical scores. Similarly, other study also found that change in physical activity was related to fewer total menopausal symptoms (β = -0.22, *P* = 0.02), psychosocial symptoms (β = -0.18, *P* = 0.05) and physical symptoms (β = -0.23, *P* = 0.01) [10].

The randomized, double-blind, placebo-controlled multicenter study has reported MENQOL score reduction of 1.6-point after hormone replacement regimen for 90 days, and the greatest difference was observed in the vasomotor domain with 3.3 point [33]. Furthermore, the randomized trials from Italy found that the reductions of MENQOL subscores between baseline and 1 year of HRT were 2.49 point for vasomotor symptoms, 0.28 point for physical symptoms, 0.45 point for psychosocial symptoms, and 0.72 point for sexual symptoms [34]. Although the variations in MENQOL scores according to the HRT appeared to exceed the magnitude of the effect of physical activity, it should be interpreted with caution because the results vary according to the conditions affecting the study populations and where the trials have been performed.

A community-based study from the United States also reported that relatively active perimenopausal women had significantly fewer and less distressing menopausal symptoms than both the active and inactive groups [3]. This nonlinear association between physical activity and menopausal symptoms may reflect the possibility that women who recognize that they are experiencing more menopausal symptoms are more likely to exercise regularly and with higher intensity. Since the present study had a cross-sectional design, further studies are required to determine the level of physical activity that is needed to reduce menopausal symptoms.

To our knowledge, this is one of few studies which investigated the association between physical activity and menopausal symptoms using validated instruments in Korean middle-aged women. However, there are some study limitations. First, the cross-sectional study design means that a causal relationship cannot be identified; thus a longitudinal study is required to achieve higher levels of evidence regarding a causal relationship. Second, all study participants were recruited from a health-screening center at two hospitals in Seoul and Suwon, which means that our results may not be generalizable to all perimenopausal women in Korea. Third, although we sought to control for potential confounding variables that could affect the menopausal symptoms, it remains possible that our results are influenced by other factors. Finally, there may have been some information bias in terms of the physical activity levels and menopausal symptoms because these variables were measured using self-reported questionnaires.

## Conclusions

In conclusion, the present study showed that moderate level of physical activity was associated with reduced psychosocial and physical menopause symptoms in perimenopausal Korean women. Although these findings must be confirmed by prospective longitudinal studies, they suggest that physical activity may improve the symptoms of menopause, thereby increasing quality of life.

## Electronic supplementary material

Additional file 1: **Prevalence of each Menopause-specific Quality of Life (MENQOL) item.** (XLSX 14 KB)

Additional file 2: **Menopause-specific Quality of Life (MENQOL) total score/subscores between the 476 participants with complete data and 155 with missing data.** (XLSX 13 KB)
